# High-density lipoprotein induces cyclooxygenase-2 expression and prostaglandin I-2 release in endothelial cells through sphingosine kinase-2

**DOI:** 10.1007/s11010-013-1941-y

**Published:** 2014-01-03

**Authors:** Sheng-Lin Xiong, Xing Liu, Guang-Hui Yi

**Affiliations:** 1 You Country People’s Hospital, Zhuzhou, 412300 Hunan China; 2 Institute of Cardiovascular Disease Research, Key Laboratory for Atherosclerology of Hunan Province, University of South China, No. 28, Changsheng Road, Hengyang, 421001 Hunan China

**Keywords:** High-density lipoprotein, Sphingosine kinase, Sphingosine 1-phosphate, Cyclooxygenase-2, Protein kinase C

## Abstract

High-density lipoprotein (HDL) has a significant cardioprotective effects. HDL induces cyclooxygenase-2 (COX-2) expression and prostacyclin I-2 (PGI-2) release in vascular endothelial cells, which contributes to its anti-atherogenic effects. However, the underlying mechanisms are not fully understood. In the present study, we observed that HDL-stimulated COX-2 expression and PGI-2 production in human umbilical vein endothelial cells (HUVECs) in a time- and dose-dependent manner. These effects triggered by HDL were inhibited by pertussis toxin (PTX), protein kinase C (PKC) inhibitor GF109203X, and ERK inhibitor PD98059, suggesting that Gαi/Gαo-coupled GPCR, PKC, and ERK pathways are involved in HDL-induced COX-2/PGI-2 activation. More importantly, we found that silencing of sphingosine kinase 2 (SphK-2) also blocked HDL-induced COX-2/PGI-2 activation. In addition, HDL-activated SphK-2 phosphorylation accompanied by increased S1P level in the nucleus. Our ChIP data demonstrated that SphK-2 is associated with CREB at the COX-2 promoter region. Collectively, these results indicate that HDL induces COX-2 expression and PGI-2 release in endothelial cells through activation of PKC, ERK1/2, and SphK-2 pathways. These findings implicate a novel mechanism underlying anti-atherothrombotic effects of HDL.

## Introduction

Epidemiological studies have shown that high plasma levels of high-density lipoproteins (HDLs) protect against development of atherosclerosis [1]. HDLs up-regulate endogenous anti-atherogenic molecules, such as prostaglandin I-2 (PGI-2), in vascular cells. PGI-2, a vasodilator that regulates vascular tone [2], also shows anti-atherogenic properties via inhibiting platelet and leukocyte activation and adhesion [3]. Several previous studies have reported that HDLs promote PGI-2 release through cyclooxygenase-2 (COX-2)-dependent mechanism [4, 5]. In addition, it has been reported that the HDL3 induces COX-2 expression and prostacyclin release in human endothelial cells via a p38 mitogen-activated protein kinase (MAPK)/CRE-dependent pathway [6]. COX-2, an enzyme responsible for inflammation and pain, has been reported to exert cardioprotective effects in a model of myocardial ischemia–reperfusion injury via activating prostaglandin (PG) E2 synthesis [7]. Collectively, these findings suggest that HDLs exert cardioprotective effects at least partially through activation of COX-2. However, the molecular mechanisms by which HDLs promote COX-2 expression in vascular endothelial cells are largely unclear.

Sphingosine 1-phosphate (S1P), a bioactive lysophospholipid, is produced by activated platelets and erythrocytes and enters circulation in HDL- or albumin-bound forms [8, 9]. In particular HDL-bound S1P has been shown to elicit diverse biological activities, including vasorelaxation [6], angiogenesis [10], endothelial cell survival and migration [11], and proliferation of vascular smooth muscle cells [12]. S1P is produced from phosphorylation of sphingosine, which is catalyzed by sphingosine kinases SphK-1 and SphK-2 [13, 14]. While SphK-1 and -2 have similar structures with five conserved domains, they differ in amino terminus and central region [15]. They also exhibit different developmental and tissue expression patterns, and have distinct kinetic properties [16, 17], which suggest that they may have a divergent physiological activities. SphK-1 regulates COX-2 expression through S1P production. Studies have shown that activation of protein kinase C (PKC) by phorbol 12-myristate 13-acetate (PMA) induces phosphorylation of SphK-1 [18]. Phosphorylated SphK-1 translocates to plasma membrane, resulting in rapid increase in S1P production and COX-2 expression in rat myometrium in late pregnancy [19]. Although SphK-2 is also activated by PKC-dependent signaling [20], the role of SphK-2 in COX-2 expression is not clear. SphK-2 is the main SphK isoform in the nucleus, which regulates nuclear S1P level. In the nucleus, S1P specifically binds to histone deacetylases HDAC1 and HDAC2 and inhibits their enzymatic activity [20]. SphK-2 is associated with HDAC1 and HDAC2 in repressor complexes and selectively enriched at promoters of genes encoding cyclin-dependent kinase inhibitor p21 and transcriptional regulator c-fos, where it enhances local histone H3 acetylation and promotes target gene transcription [20]. While SphK-1 promotes growth and survival, SphK-2 suppresses growth and enhances apoptosis [21]. Considering that SphK-1 regulates COX-2 expression via S1P production, we speculated that SphK-2 might play a role in HDL-induced COX-2 expression in vascular endothelial cells via activating S1P synthesis in the nucleus. In this study, we investigated whether HDL regulates COX-2 expression through SphK-2-dependent pathway in human umbilical vein endothelial cells (HUVECs). Moreover, since cAMP response element-binding (CREB) protein, a key transcription factor, is involved in transcriptional activation of COX-2 expression [22], we also investigated the interaction between CREB and SphK-2.

## Materials and methods

### Materials

[γ-^32^P]ATP (3,000 Ci/mmol) and [^32^P]orthophosphate were purchased from PerkinElmer Life Sciences (Turku, Finland). HUVECs were purchased from the Medical Center for Cells, Central South University (Changsha, China). Pertussis toxin (PTX), NS398, and 4′-6-diamidino-2-phenylindole were obtained from Sigma (St. Louis, USA). GF109203X, PD98059, JTE013, and VPC23019 were from Cayman (Michigan, USA). Competitive enzyme immunoassay kit for 6-keto PGF1α, nuclear and cytoplasmic protein extraction kit, and chromatin immunoprecipitation (ChIP) assay kit were obtained from Beyotime Institute of Biotechnology (Haimen, China). Mouse GAPDH was from Boster (Wuhan, China). Rabbit anti-COX-2 antibodies were from Epitomics (Burlingame, USA). Rabbit polyclonal anti-SphK-2 and anti-SphK1 antibodies were from Abcam (Cambridge, USA). P-CREB, ERK1/2, P-ERK1/2, P-PKCα, and PKCα antibodies were from Cell Signaling (Boston, USA). HDL was purchased from Millipore Corporation (Billerica, USA). Lipofectamine 2000 was from Invitrogen (Carlsbad, USA). The small interfering RNA (siRNA) for *SphK*-*2* was obtained from Ruibo Co., Ltd. (Guangzhou, China). RealSuper Mixture (with ROX) was obtained from Cowin Biotech Co., Ltd. (Beijing, China).

### Cell culture and labeling of cellular SphK-2

HUVECs were cultured in Dulbecco’s Modified Eagle Medium (DMEM) supplemented with 10 % fetal bovine serum, 100 U/ml of penicillin, and 100 mg/L of streptomycin at 37 °C, 5 % CO_2_ in a humidified incubator. The medium was changed every 3 days until cells were confluent. Cells were then incubated for 6 h in serum-free medium before transfection. Transfected HUVECs were serum-starved overnight in phosphate-free DMEM, metabolically labeled by incubation in medium containing [^32^P]orthophosphate (70 μCi/ml) at 37 °C for 4 h, and subsequently treated with PTX (100 ng/ml, 24 h), JTE013 (10 μmol/L, 1 h), VPC23019 (10 μmol/L, 1 h), PD98059 (10 μmol/L, 1 h), GF109203X (10 μmol/L, 1 h), or their respective vehicle controls. Cells were then washed once with 4-(2-hydroxyethyl)-1-piperazineethanesulfonic acid-buffered medium and stimulated with HDL.

### Quantitation of sphingolipids

Cytoplasm and nuclei were isolated using a Nuclear and Cytoplasmic Protein Extraction Kit (Beyotime Biotechnology, Hangzhou, China) according to the manufacturer’s instructions. Lipids were extracted and sphingolipids were determined by liquid chromatography coupled with electrospray ionization–tandem mass spectrometry (LC–ESI–MS/MS) using LIPID MAPS™ Incinerator calendar provided by Onyalai Lipids (Alabaster, AL). Internal standards were added in 0.5-fold quantities.

### SphK-2 activity

Cells were lysed by repeated freeze and thaw cycles. SphK-2 activity in cell lysates was determined in the presence of 1 M KCl, under which SphK-2 exhibits optimal activity, while SphK-1 is strongly inhibited [23]. In brief, cell lysates were incubated with 50 μM sphingosine [in the form of complex with bovine serum albumin (BSA)] and [γ-^32^P]ATP at 37 °C for 30 min. Reactions were stopped by the addition of 500 μl of chloroform/methanol/concentrated HCl (100:100:1, vol/vol). After addition of 100 μl chloroform and 100 μl 2 M KCl for phase separation, samples were vortexed and centrifuged at 700×*g* for 5 min. Aliquots (100 μl) of organic phase containing labeled lipids were separated on TLC using a mobile phase of chloroform/acetone/methanol/acetic acid/water (50:20:15:10:5, vol/vol).

### siRNA transfection

HUVECs were plated in six-well plates (1 × 10^6^ cells/well). At 30–50 % confluence, HUVECs were transfected with 50 or 100 nM of *SphK*-*2* siRNA or scrambled control siRNA in OptiMEM (Invitrogen, CA, USA) using Lipofectamine 2000 according to manufacturer’s protocol. Target sequences for human *SphK*-*2* siRNA were 5-CAAGGCAGCUCUACACUCA-3 (sense) and 3-GUUCCGUCGAGAUGUGAGU-5 (antisense), and those for scrambled control siRNA were 5-CGGCCUACUUCUGCAUCUA-3 (sense) and 3-GCCGGAUGAAGACGUAGAU-5 (antisense). After 24 h, the medium was replaced with fresh serum-free DMEM. After another 48 h incubation, HUVECs were treated with 120 μg/ml of HDL for 8 h. COX-2 expression and PGI-2 release were determined.

### Quantitation of 6-keto PGF1α by competitive ELISA

To measure 6-keto-PGF1α production, HUVECs were treated with HDL (120 μg/ml) for 8 h, washed twice with phosphate buffered saline (PBS), and incubated in PBS buffer containing 0.75 % fatty acid-free BSA and exogenous AA (10 μmol/L) at 37 °C, 5 % CO2 for 30 min in a humidified incubator [6]. PGI-2 release in samples (50 μl each) was determined according to manufacturer’s instructions.

### Protein extraction

Nuclear and cytoplasmic proteins were extracted using cell lysis buffer (Beyotime, Haimen, China) according to manufacturer’s instructions.

### Protein analysis by Western blotting

Equal amounts of proteins were separated by 10 % sodium dodecyl sulfate-based polyacrylamide gel electrophoresis (SDS–PAGE), and transferred to polyvinylidenedifluoride (PVDF) membranes. Membranes were incubated with COX-2, SphK-2, P-CREB, P-PKCα, PKCα, P-ERK1/2, ERK1/2, and GAPDH antibodies, respectively, washed, and incubated with anti-rabbit or anti-mouse secondary antibodies conjugated with horseradish peroxidase for detection. Immunopositive bands were visualized by enhanced chemiluminescence and quantified using a densitometer (Molecular Dynamics, Sunnyvale, CA, USA).

### Real-time PCR

Total RNA was isolated using TRIzol reagent (Sigma, St. Louis, USA) and reverse transcribed by random priming and Moloney Murine leukemia virus reverse transcriptase (Fermantas, Ontario, Canada) according to manufacturer’s instructions. Primers specific for human *COX*-*2* and *GAPDH* were obtained from Invitrogen (Shanghai, China) and had sequences as follows: *COX*-*2*: 5′-TTC AAA TGA GAT TGT GGG AAA ATT GCT-3 (sense), 5′-AGA TCA TCT CTG CCT GAG TAT CTT-3′ (antisense); *GAPDH*: 5′-CAA GGT CAT CCA TGA CAA CTT TG-3′ (sense), 5′-GTC CAC CAC CCT GTT GCT GTA G-3′ (antisense). mRNA expression levels of *COX*-*2* and *GAPDH* were quantified using SYBR Green Master Mix (with ROX) on a real-time PCR detection system (Applied Biosystems, CA, USA). mRNA levels of *COX*-*2* were normalized to *GAPDH.*

### Immunoprecipitation assays

HUVEC cells cultured for 48 h were harvested by scraping and lysed in 1 ml lysis buffer (50 mM Tris–HCl, 75 mM NaCl, 75 mM KCl, 4 mM MgCl_2_, 1 % NP-40) containing 1X Halt Protease Inhibitor Cocktail (Pierce, Rockford, IL, USA) [24]. Lysate (500 μg of total protein) was subjected to immunoprecipitation by incubation with 10 μg of rabbit polyclonal SphK-1 antibody or rabbit polyclonal SphK-2 antibody in the presence of Protein A+G agarose beads. Beads were boiled in Laemmli sample buffer to elute precipitated proteins [24]. Precipitated protein samples were then separated on 10 % SDS-PAGE gels and transferred to PVDF membranes. Membranes were incubated with rabbit anti-p-CREB antibody, rabbit polyclonal SphK-1 antibody, or rabbit polyclonal SphK-2 antibody, washed, and incubated with anti-rabbit secondary antibody conjugated with horseradish peroxidase for detection.

### Chromatin immunoprecipitation (ChIP)

ChIP was conducted using a ChIP assay kit from Beyotime (Haimen, China) with slight modifications. Immunoprecipitated DNA was purified and amplified across the *COX*-*2* promoter region using 5′ primer ^−119^TAA GGG GAG AGG AGG GAA AAA T^−97^ and 3′ primer ^+6^ACA ATT GTC GCT AAC CGA G^−14^ [25].

### Statistical analysis

Data are presented as mean ± SE. Statistical analysis was performed using Student’s *t* test or ANOVA analysis. Differences with *P* < 0.05 were considered statistically significant.

## Results

### HDL induces COX-2 expression and PGI-2 release in HUVECs

**Fig. 1:**
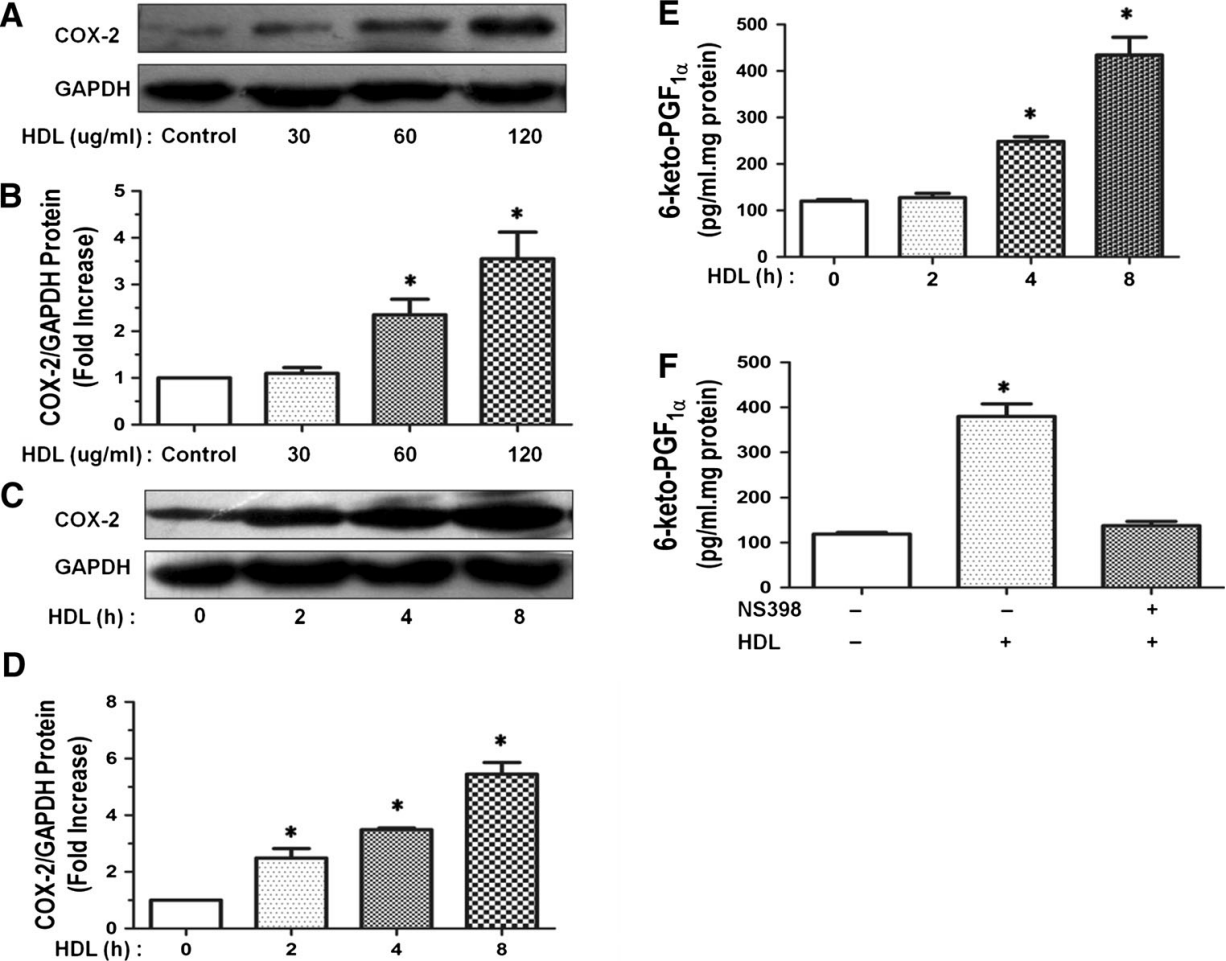
HDL-induced COX-2 expression and PGI-2 release in HUVECs. **a**, **b** HUVECs were incubated with PBS (Control group), or HDL. HUVECs were incubated with different doses of HDL for 8 h, and the expression of COX-2 was assayed by Western blot analysis (**a**). The relative protein expression was normalized to GAPDH (**b**). **c**, **d** HUVECs were incubated with PBS or HDL (120 μg/ml) at different time points, and the expression of COX-2 was assayed by Western blot analysis (**c**). The relative protein expression was normalized to GAPDH (**d**). **e** HUVECs were incubated with PBS or HDL (120 μg/ml) at different time points, the production of PGI-2 was determined by competitive ELISA (**e**). **f** The cells were preincubated for 60 min with or without 10 μM NS398 and then stimulated by HDL (120 μg/ml). The production of PGI-2 was determined by competitive ELISA (**f**). Data were mean ± SE from three separate experiments. *P < 0.05 versus Control group

Vascular endothelial cells play an important role in many pathological and physiological processes and are extensively studied in medical research. HUVECs resemble arterial endothelial cells and are widely used in studies of vascular endothelial cells, since they are more easily available than cells from many other vessels [26]. Since several previous reports have indicated that HDL activates PGI-2 release through COX-2-dependent mechanisms [4, 27], we investigated that the effects of HDL on COX-2 expression and PGI-2 release in HUVECs. As shown in Fig. 1a–d, HDL-induced COX-2 protein expression in HUVECs in a dose (60–120 μg/ml)- and time (2–8 h)-dependent manner. Maximal COX-2 expression was observed after 8 h of incubation with 120 μg/ml of HDL. Similarly, HDL-activated PGI-2 release into culture medium in a time-dependent manner, presumable by increasing COX-2-catalyzed PGI-2 production (Fig. 1e). Indeed, HDL-induced PGI-2 release was attenuated by NS398, a specific inhibitor of COX-2 (Fig. 1f). Therefore, our results showed that HDL activates PGI-2 release via inducing COX-2 expression in HUVECs. Consequently, we investigated that the molecular mechanisms underlying HDL-induced COX-2 expression and PGI-2 release.

### HDL induces COX-2 expression and PGI-2 release via PTX-sensitive Gαi/Gαo proteins

**Fig. 2:**
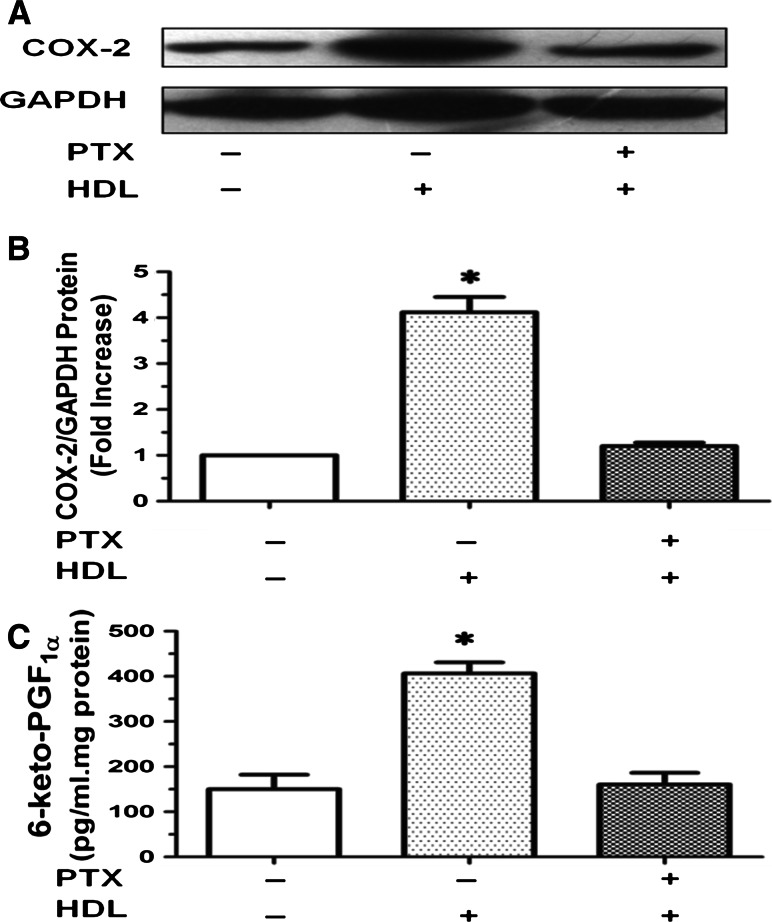
Effect of PTX on the HDL-induced COX-2 expression and PGI-2 release. **a**–**c** Cells were washed and pretreated with PBS or 100 ng/ml PTX for 24 h and then stimulated without or with 120 μg/ml HDL for 8 h. The expression of COX-2 was assayed by Western blot analysis (**a**), relative protein expression of COX-2 was normalized to GAPDH, and the production of PGI-2 was determined by competitive ELISA (**c**). Data were mean ± SE from three separate experiments. *P < 0.05 versus Control group

We postulated that S1P receptors coupled to PTX-sensitive Gαi/Gαo cells with PTX. Our data showed that incubation of HUVECs with PTX, a specific inhibitor of Gαi/Gαo-protein, prior to HDL treatment markedly attenuated HDL-induced COX-2 expression (Fig. 2a, b) and PGI-2 release (Fig. 2c). We, therefore, speculated that S1P receptors coupled to PTX-sensitive Gαi/Gαo proteins may mediate the effects of HDL.

### HDL-induced COX-2 expression and PGI-2 release are PKC- and ERK-dependent

**Fig. 3:**
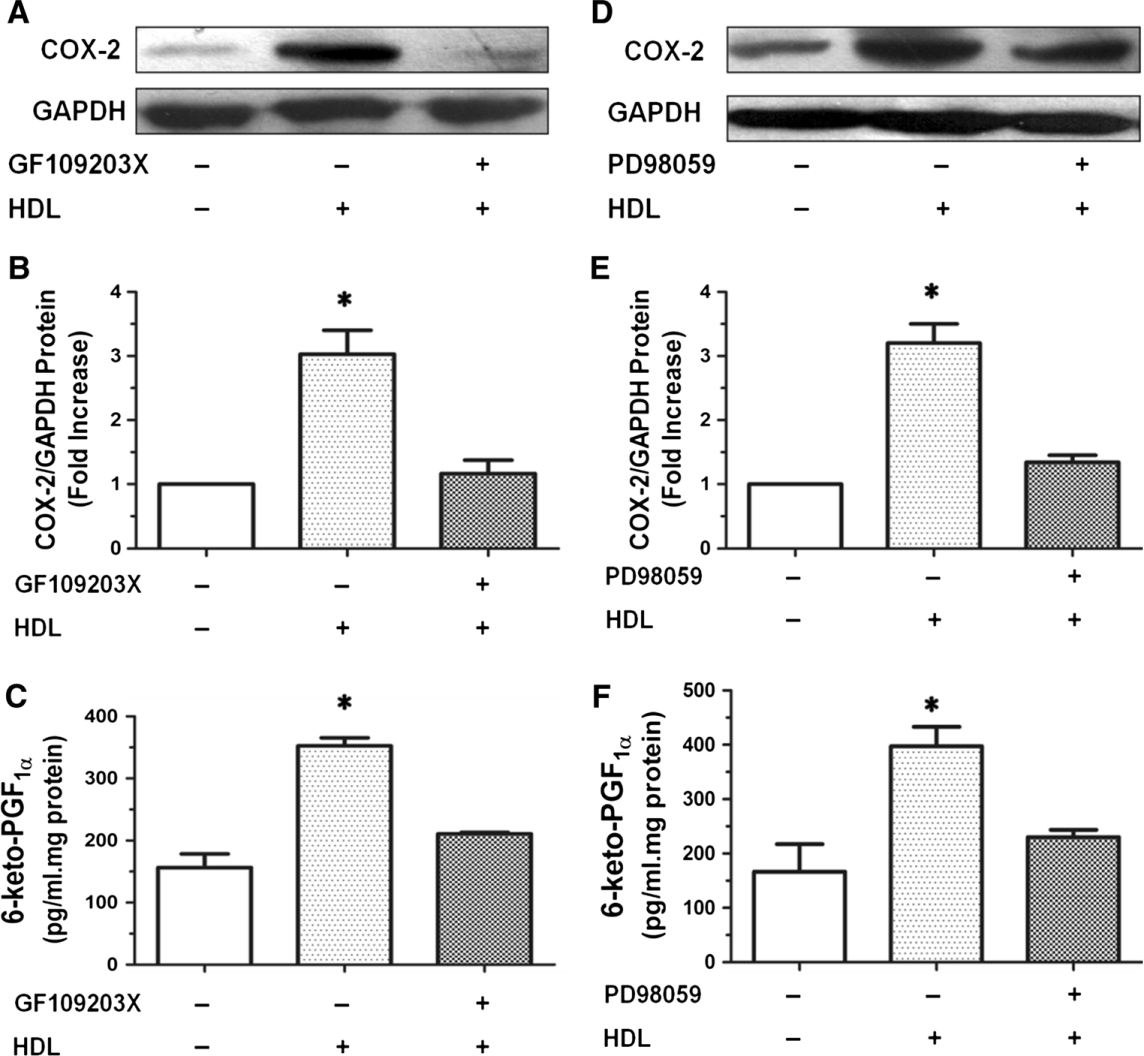
COX-2 expression and PGI-2 release induced by HDL were inhibited by GF109203X or PD98059 in HUVECs. **a**–**c** Cells were washed and pretreated with PBS or the PKC inhibitor GF109203X (10 μmol/L) for 1 h, and then stimulated without or with 120 μg/ml HDL for 8 h. The expression of COX-2 was assayed by Western blot analysis (**a**), relative protein expression of COX-2 was normalized to GAPDH (**b**), and the production of PGI-2 was determined by competitive ELISA (**c**). **d**, **e** Cells were washed and pretreated with PBS or the extracellular regulated kinase (ERK)1/2 inhibitor PD98059 (10 μmol/L) for 1 h, and then stimulated without or with 120 μg/ml HDL for 8 h. The expression of COX-2 was assayed by Western blot analysis (**d**), relative protein expression of COX-2 was normalized to GAPDH (**e**), and the production of PGI-2 was determined by competitive ELISA (**f**). Data were mean ± SE from three separate experiments. *P < 0.05 versus Control group

Our results also showed that COX-2 expression and PGI-2 release triggered by HDL were inhibited by GF109203X, a specific PKC inhibitor (Fig. 3a–c) and PD98059, a ERK kinase inhibitor (Fig. 3d–f), indicating that PKC- and ERK-dependent pathways are involved in HDL-induced COX-2 expression and PGI-2 release in HUVECs.

### HDL-induced COX-2 expression and PGI-2 release are accompanied by PKCα and ERK1/2 activation

**Fig. 4:**
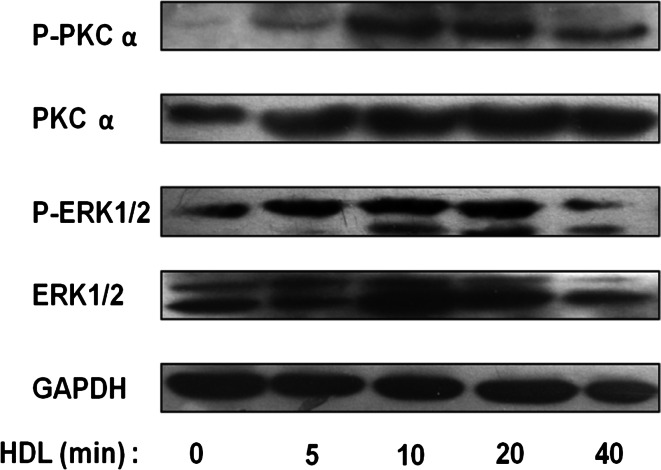
Phosphorylation of signaling pathways involved in COX-2 expression and PGI-2 release induced by HDL in HUVECs. **a** HUVECs were incubated with 120 μg/ml of HDL at different time points, and the activation of PKCα and ERK1/2 was analyzed by Western blot analysis. Protein samples were immunoblotted with anti-phospho-PKCα, anti-phospho-ERK1/2, anti-total-PKCα, anti-total-ERK1/2 and anti-GAPDH antibodies

HUVECs were treated with 120 μg/ml of HDL for different durations, and activation of PKCα and ERK1/2 was detected by Western blot analysis of protein phosphorylation (Fig. 4a). Our results showed that HDL-activated PKCα and ERK1/2 in a time-dependent manner, with maximal phosphorylation observed at 20 min. Our Western blotting data further supported that HDL promotes COX-2 expression and PGI-2 release in HUVECs via activating PKCα- and ERK1/2-dependent pathways.

### HDL induces COX-2 expression and PGI-2 release via activation of S1P receptors (S1PRs)

**Fig. 5:**
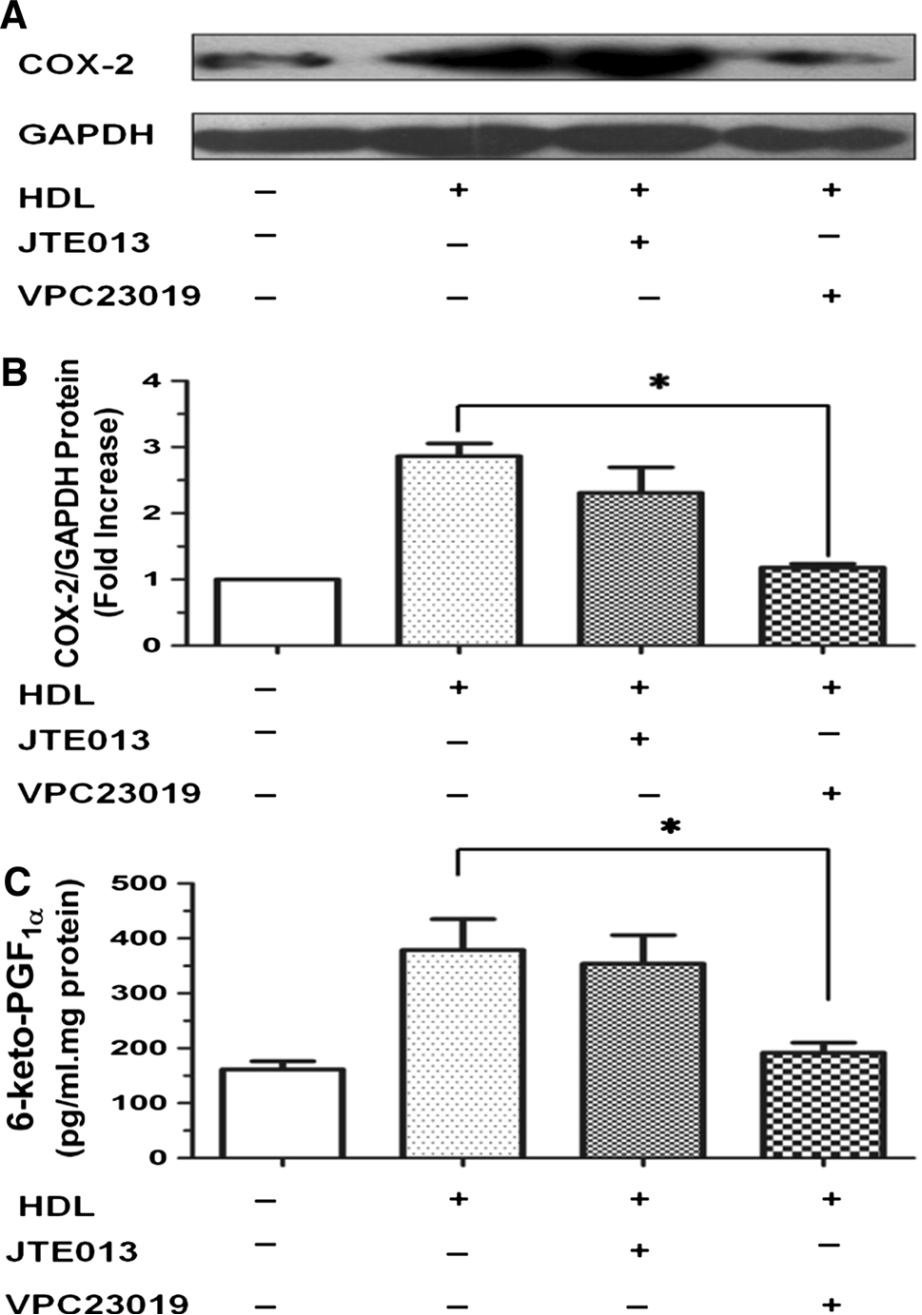
COX-2 expression and PGI-2 release induced by HDL were inhibited by different S1P receptors (S1PRs) inhibitors in HUVECs. **a**–**c** Cells were washed and pretreated with PBS or the S1P1 and S1P3 inhibitor VPC23019 (10 μmol/L), or the S1P2 inhibitor JTE013 (10 μmol/L) for 1 h, and then stimulated without or with 120 μg/ml HDL for 8 h. The expression of COX-2 was assayed by Western blot analysis (**a**), relative protein expression of COX-2 was normalized to GAPDH (**b**),and the production of PGI-2 was determined by competitive ELISA (**c**). Data were mean ± SE from three separate experiments. *P < 0.05 versus VPC23019/HDL group

S1P receptors are present in the cardiovascular system. S1P receptors 1–3 (S1PR1–3) are expressed in endothelial cells, whereas S1PR1–4 are expressed in myocardial tissues [28]. We found that HDL-induced COX-2 expression and PGI-2 release were inhibited by pretreatment with VPC23019, an inhibitor of S1PR1 and S1PR3, but hardly affected by JTE, an inhibitor of S1PR2 (Fig. 5a–c). These results suggested that HDL induces COX-2 expression and PGI-2 release in HUVECs via activation of S1PR1 and S1PR3.

### SphK-2 silencing inhibits HDL-induced COX-2 expression and PGI-2 production

**Fig. 6:**
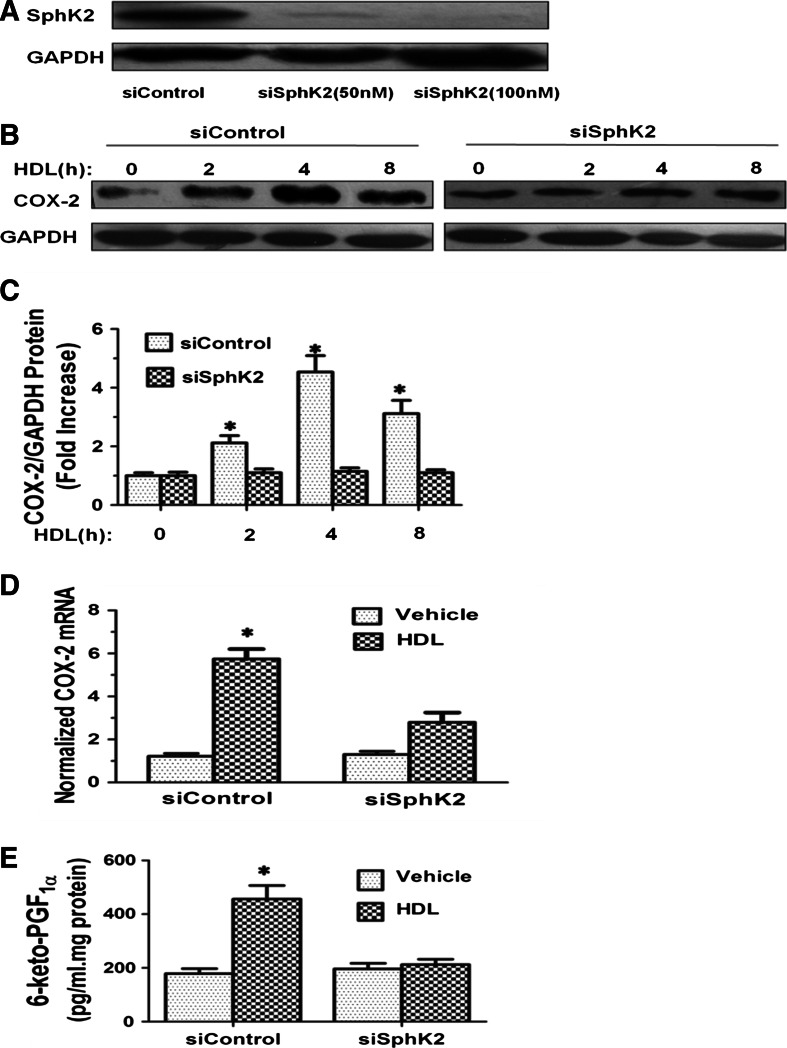
Knockdown of SphK-2 reduces COX-2 induction and PGI-2 release by HDL in HUVECs. Cells were transfected with 50 or 100 nM siRNA of SphK-2. The expression of SphK-2 was assayed by Western blot analysis (**a**). 48 h after siControl and siSphK-2 (100 nM) transfection, cells were treated with PBS or HDL (120 μg/ml) for 8 h. The expression of COX-2 was assayed by Western blot analysis (**b**), relative protein expression of COX-2 was normalized to GAPDH (**c**), COX-2 mRNA levels were assayed by quantitative real-time PCR (**d**), and the production of PGI-2 was determined by competitive ELISA (**e**). Data were mean ± SE from three separate experiments. *P < 0.05 versus siControl group

To investigate whether SphK-2 is involved in HDL-induced COX-2 expression and PGI-2 production, we studied the effect of *SphK*-*2* silencing by siRNA transfection. Western blot analysis showed that SphK-2 protein level was much lower in HUVECs transfected with *SphK*-*2* specific siRNA than those transfected with control siRNA (Fig. 6a). HUVECs transfected with *SphK*-*2* specific siRNA or control siRNA were serum-starved overnight in phosphate-free DMEM, washed, and treated with 120 μg/ml HDL or vehicle for different durations. Cells were then lysed and intracellular levels of COX-2 and PGI-2 were determined by Western blot analysis and competitive ELISA, respectively, as described in Materials and Methods. Our results showed that *SphK*-*2* silencing abrogated HDL-induced COX-2 expression (Fig. 3b–d) and PGI-2 production (Fig. 3e). These data demonstrated that SphK-2 mediates HDL-induced COX-2 expression and PGI-2 production.

### HDL activates SphK-2 and promotes PGI-2 release via Gαi/Gαo-, PKC-, and ERK-dependent pathways

**Fig. 7:**
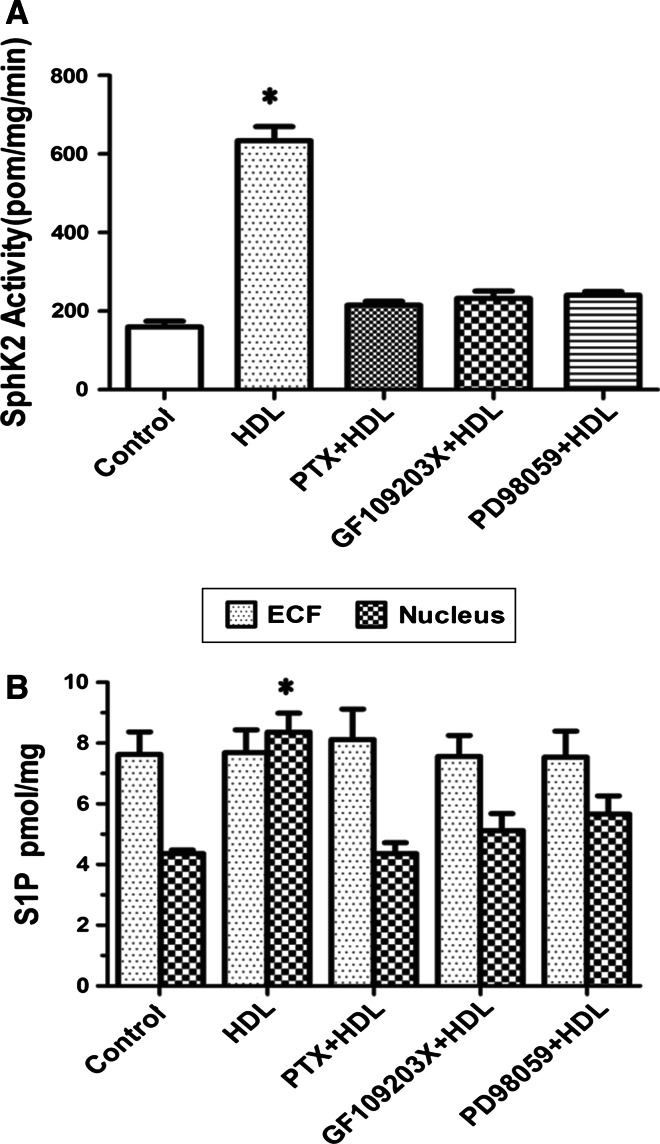
Phosphorylation of SphK-2 and PGI-2 release induced by HDL were inhibited by different inhibitors in HUVECs. HUVECs were serum-starved overnight in phosphate-free DMEM and metabolically labeled with ^32^Pi for 4 h prior to treatment with 100 ng/ml PTX for 24 h, 10 μmol/L GF109203X for 1 h, 10 μmol/L PD98059 for 1 h,and then stimulated with PBS or with 120 μg/ml HDL for 20 min. Cell lysates were immunobloted with ^32^P incorporation determined by autoradiography. SphK-2 activity in the immunoblotes was determined (**a**). Lipids were extracted, the S1P level was determined by LC–ESI–MS/MS (**b**). Data were mean ± SE from three separate experiments. *P < 0.05 versus PTX/HDL, GF109203X/HDL, PD98059/HDL group

The PKC activator, PMA, has been shown to stimulate SphK-1 by inducing its phosphorylation [18], which is not only required for translocation of SphK-1 to the plasma membrane but also for activation of *COX*-*2* gene expression [18, 19]. In contrast, mechanisms of SphK-2 activation remain largely unknown. Considering that SphK-1 and SphK-2 are highly homologous, we investigated whether SphK-2 is also activated by phosphorylation. HUVECs were serum-starved overnight in phosphate-free DMEM, metabolically labeled with ^32^P, incubated with PTX, GF109203X, or PD98059, and then treated with 120 μg/ml HDL or vehicle for 20 min. Cell lysates were immunoblotted with SphK-2 antibody and ^32^P incorporation was determined by autoradiography. SphK-2 activity in immunoprecipitates was also determined. We found that SphK-2 was constitutively phosphorylated, and ^32^P incorporation was markedly increased after treatment with HDL. Moreover, HDL-induced SphK-2 phosphorylation was accompanied by significantly increased SphK-2 activity in immunoprecipitates. Treatment of cells with PTX, GF109203X, or PD98059 markedly inhibited HDL-stimulated increase in SphK-2 activity (Fig. 7a). In addition, level of S1P in the nucleus increased after treatment with HDL. Incubation with PTX, GF109203X, or PD98059 markedly attenuated HDL-induced increase in nuclear S1P concentration. In contrast, S1P level in extracellular fluid was not affected by HDL treatment (Fig. 7b). Collectively, these data provided further evidence that HDL stimulates PGI-2 release by activating SphK-2, which acts downstream of Gαi/Gαo-, PKC-, and ERK in HDL-induced signal transduction.

### SphK-2 interacts with CREB

**Fig. 8:**
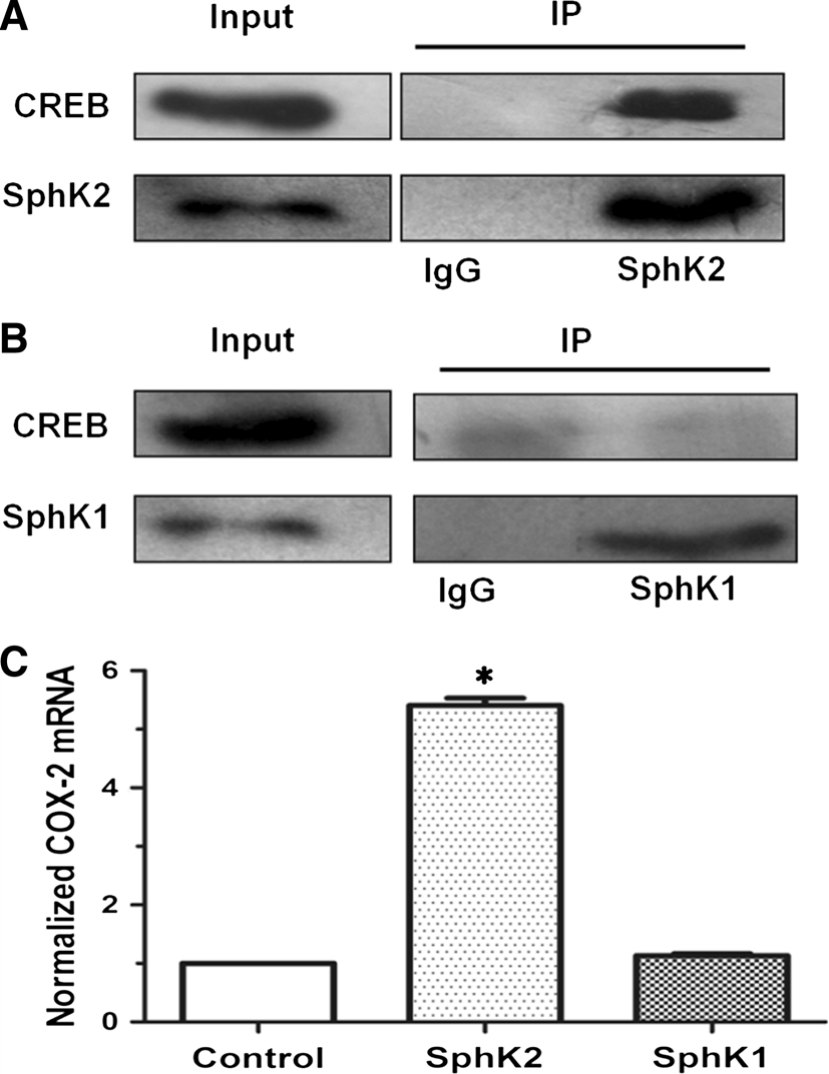
SphK-2 interacts with CREB. Cells were pulled down as described under experimental procedures. HUVECs were incubated with anti-SphK-2 or IgG antibodies coupled to agarose beads.Bound proteins were analyzed by Western blotting with anti-CREB and SphK-2 antibodies. 10 μg of total cell lysates from each sample were also analyzed by immunoblotting with anti-CREB and SphK-2 antibodies as inputs (**a**). HUVECs were incubated with anti-SphK-1 or IgG antibodies coupled to agarose beads. Bound proteins were analyzed by Western blotting with anti-CREB and SphK-1 antibodies. 10 μg of total cell lysates from each sample were also analyzed by immunoblotting with anti-P-CREB and SphK-1 antibodies as inputs (**b**). HUVEC cells incubated with anti-SphK1, or SphK2 antibodies coupled to agarose beads, then treated with vehicle or HDL (120 mg/ml) for 20 min and mRNA levels of COX-2 were determined by qPCR and normalized to GAPDH. **c** Results from at least three independent experiments conducted in triplicate are shown. *P < 0.05, versus cells treated with SphK-1 antibodies group

CREB protein, a key transcription factor and downstream effector of PKC, regulates *COX*-*2* transcription [29]. Although recent reports have indicated that phorbol esters, activators of PKC, also stimulate SphK-2 activity [20], there is no evidence that CREB and SphK-2 interacts with each other. We found that SphK-2 in vascular endothelial cells contributes to S1P production in the nucleus (Fig. 7b). In the nucleus, S1P specifically binds to histone deacetylases HDAC1 and HDAC2 and inhibits their enzymatic activity, resulting in activation of target gene transcription [20]. Whether SphK-2 activates *COX*-*2* gene transcription via increasing nuclear S1P level is not clear either. To find out whether there is a direct interaction between CREB and SphK-2, we conducted immunoprecipitation experiments. We found that specific pull down of SphK-2 brought down CREB, but not SphK-1, suggesting that CREB is associated with SphK-2 (Fig. 8a, b). Furthermore, we studied contribution of SphK-1 and SphK-2 to HDL-induced *COX*-*2* gene expression using ChIP. HUVECs were treated with HDL (120 mg/ml) for 20 min and then incubated with anti-SphK-2 antibody or anti-SphK-1 antibody coupled to agarose beads. Results from ChIP showed that HDL treatment significantly increased SphK-2-associated, but not SphK-1-associated *COX*-*2* promoter activity (Fig. 8c), in agreement with involvement of SphK-2 in HDL-induced gene expression of *COX*-*2*.

## Discussion

HDL, arguably the most potent anti-atherogenic factor in humans, independently predicts epidemiologic risk of cardiovascular events [30]. HDL cholesterol via promoting reverse cholesterol transport. In addition, HDL has other potent anti-atherogenic properties including anti-inflammatory, anti-oxidative, anti-apoptotic, and nitric oxide (NO)-generating activities [1, 31, 32]. The mechanisms by which HDL exerts its powerful anti-atherogenic effects are not entirely understood.

Numerous studies have shown that COX-2, a key enzyme in PG synthesis, is a downstream effector of a variety of extracellular signaling molecules, including G-protein-coupled receptor (GPCR) ligands and cytokines [33, 34]. Our results showed that HDL significantly upregulates *COX*-*2* expression and PGI-2 release in HUVECs (Fig. 1). These effects of HDL were abrogated by PTX, an irreversible inhibitor of Gαi/Gαo proteins (Fig. 2), GF109203X, a PKC inhibitor, and PD98059, an ERK inhibitor (Fig. 3), suggesting that Gαi/Gαo-coupled GPCR, PKC, and ERK pathways are involved in HDL-induced COX-2/PGI-2 activation. In particular, our results are in agreement with previous reports that p38 MAPK and ERK1/2 are involved in upregulation of *COX*-*2* gene expression by HDL [6, 35]. We also observed that HDL treatment induced phosphorylation of PKC and ERK1/2 (Fig. 4), which provided further evidence that PKC and ERK1/2 mediate effects of HDL. Collectively, these findings suggest that HDL protects endothelial cells through activation of *COX*-*2*/PGI-2 signaling via PKC and ERK1/2 pathways.

It has been reported that HDL binds to and activates S1PR1–3, which are Gαi/αo-coupled GPCRs, leading to activation of intracellular signaling pathways and COX-2 expression [36]. In this study, we observed that, while VPC23019, an inhibitor of S1PR1 and S1PR3 significantly attenuated HDL-induced *COX*-*2*/PGI-2 activation, JTE013, an inhibitor of S1P2 did not show significant effect (Fig. 5). These results suggest that effects of HDL are mainly mediated by S1PR1 and S1PR3 rather than S1PR2.

**Fig. 9:**
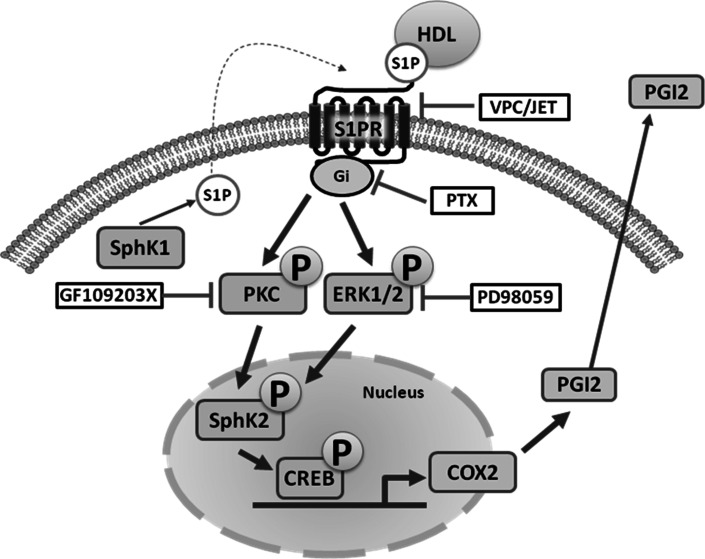
S1PR/PKC/ERK/SphK signaling pathways involved in COX-2 expression and PGI-2 release induced by HDL in HUVECs. SphK-2 signaling pathways involved in HDL-induced COX-2 expression and PGI-2 release in HUVECs. Binding of HDL-S1P to S1P receptors resulted transactivation of PKC/ERK linking to SphK-2. The COX-2 transcription is dependently regulated by SphK-2 interaction with CREB. These signaling pathways contribute to COX-2 expression and PGI-2 release in HUVECs. Speculated that the activation of SphK-1 which increases the cytosolic pool of S1P leading to cellular release and the activation of S1P receptors on the cell surface

In the nucleus, S1P activates target gene expression by inhibiting histone deacetylases HDAC1 and HDAC2 [20]. Although SphK-1 and SphK-2 both contribute to S1P production, SphK-2 is the dominant isoform in the nucleus [37]. SphK-1 has been reported to act downstream of PKC and mediates phorbol ester-stimulated COX-2 expression. Although there is no direct evidence that SphK-2 regulates COX-2 expression, SphK-2 may be activated by ERK [38]. In this study, we investigated the role of SphK-1 and SphK-2 in HDL-induced COX-2/PGI-2 activation. We found that SphK-2 silencing nearly abolished HDL-induced *COX*-*2* expression and PGI-2 release in HUVECs (Fig. 6). Furthermore, HDL-induced phosphorylation of SphK-2, which was accompanied by increased S1P level in the nucleus (Fig. 7). CREB, a key transcription factor, regulates *COX*-*2* transcriptional [39]. However, the relation between CREB and SphK-2 remains unclear. Our ChiP data showed that SphK-2 is associated with CREB at the COX-2 promoter region (Fig. 8). Collectively, these results suggest that HDL stimulates *COX*-*2* transcription in HUVECs via SphK-2/S1P activation in the nucleus. In addition, we found that specific inhibitors of Gαi/αo, ERK1/2, and PKC markedly reduced HDL-induced phosphorylation of SphK-2 (Fig. 7), suggesting that SphK-2 acts downstream of Gαi/αo, ERK1/2, and PKC pathways in HDL-stimulated signal transduction. Figure 9 is a schematic diagram of HDL-activated signaling pathways leading to *COX*-*2* expression and PGI-2 release in HUVECs.

## Conclusions

In summary, our results provide the first evidence that HDL induces COX-2 expression and PGI-2 release by SphK-2 phosphorylation and activation. The significance of SphK-2 in cardioprotective effects of HDL and in lipid metabolism needs to be further investigated.

## Abbreviations

HDL: High-density lipoprotein
SphK: Sphingosine kinase
S1P: Sphingosine 1-phosphate
COX-2: Cyclooxygenase-2
PGI-2: Prostacyclin I-2
PKC: Protein kinase C
ERK1/2: Extracellular receptor kinase 1/2
HUVEC: Human umbilical vein endothelial cell
GAPDH: Glyceraldehyde-3-phosphate dehydrogenase
6-keto PGF1α: 6-Keto-prostaglandin F1α
PGI-2: Prostacyclin I-2
